# Net ecosystem production and carbon dioxide fluxes in the Scheldt estuarine plume

**DOI:** 10.1186/1472-6785-8-15

**Published:** 2008-09-08

**Authors:** Alberto V Borges, Kevin Ruddick, Laure-Sophie Schiettecatte, Bruno Delille

**Affiliations:** 1 University of Liège, Chemical Oceanography Unit, Institut de Physique (B5), B-4000, Liège, Belgium; 2 Management Unit of the North Sea Mathematical Models, Royal Belgian Institute for Natural Sciences, 100 Gulledelle, B-1200, Brussels, Belgium

## Abstract

**Background:**

A time series of 4 consecutive years of measurements of the partial pressure of CO_2 _(pCO_2_) in the Scheldt estuarine plume is used here to estimate net ecosystem production (NEP).

**Results:**

NEP in the Scheldt estuarine plume is estimated from the temporal changes of dissolved inorganic carbon (DIC). The strong seasonal variations of NEP are consistent with previous reports on organic carbon dynamics in the area. These variations are related to successive phytoplankton blooms that partly feed seasonally variable heterotrophy the rest of the year. On an annual time scale the Scheldt estuarine plume behaves as a net heterotrophic system sustained with organic carbon input from the Scheldt inner estuary and the Belgian coast. During one of the years of the time-series the estuarine plume behaved annually as a net autotrophic system. This anomalous ecosystem metabolic behaviour seemed to result from a combination of bottom-up factors affecting the spring phytoplankton bloom (increased nutrient delivery and more favourable incoming light conditions). This net autotrophy seemed to lead to a transient aa accumulation of organic carbon, most probably in the sediments, that fed a stronger heterotrophy the following year.

**Conclusion:**

The present work highlights the potential of using pCO_2 _data to derive detailed seasonal estimates of NEP in highly dynamic coastal environments. These can be used to determine potential inter-annual variability of NEP due to natural climatic oscillations or due to changes in anthropogenic impacts.

## Background

The flows of carbon and nutrients in the coastal ocean are disproportionately high in comparison with its surface area because of the massive inputs of organic matter and nutrients from land. Large amounts of matter and energy are exchanged between the coastal ocean and the open ocean across continental slopes and the coastal ocean represents one of the most biogeochemically active areas of the biosphere [e.g., [1]]. The production, degradation, export and burial of organic matter in coastal waters are in general much higher than in the open ocean [e.g., [2]].

The metabolic status of an ecosystem is quantified by the net ecosystem production (NEP) that corresponds to the difference between gross primary production (GPP) and ecosystem respiration (autotrophic and heterotrophic respiration) in both the pelagic and benthic compartments. This will determine if an ecosystem exports organic carbon to adjacent systems (net autotrophic; NEP > 0) or if an ecosystem requires external organic carbon inputs to sustain its ecosystem metabolism (net heterotrophic; NEP < 0). However, the ecosystem metabolic status of the coastal ocean as net autotrophic or net heterotrophic has been the subject of a long lived debate [1-6]. One of the reasons for this debate is the lack of data for resolving the temporal variability of carbon cycling in the highly dynamic coastal ecosystems, and for adequately describing the diversity and spatial heterogeneity of these ecosystems [1,7-9]. A recent exhaustive literature review of ecosystem metabolic estimates in European coastal waters did not reach an unambiguous conclusion on their trophic status, although these are among the most thoroughly studied sites in the world [7].

Reliable estimates of the ecosystem metabolic status are hampered by the conceptual problems associated with ^14^C estimation of primary production [e.g., [10,11]], the strong spatial heterogeneity within an ecosystem [e.g., for estuaries [12,13]], and the high temporal variability [e.g., [14]] which cannot be easily measured with classical incubation based approaches. Gazeau et al. [12] reviewed the advantages and caveats of several methods to estimate NEP, and recommended the use of integrative mass balance approaches. A commonly applied integrative mass balance approach is the Land-Ocean Interaction in the Coastal Zone (LOICZ) method based on the budget of dissolved inorganic phosphorus (DIP) [15]. In turbid environments such as inner and outer estuaries, the LOICZ DIP budgets can provide highly unrealistic NEP estimates [e.g., [12,13]] due to complex abiotic processes of desorption/adsorption from/on suspended matter [e.g., [16]].

**Figure 1:**
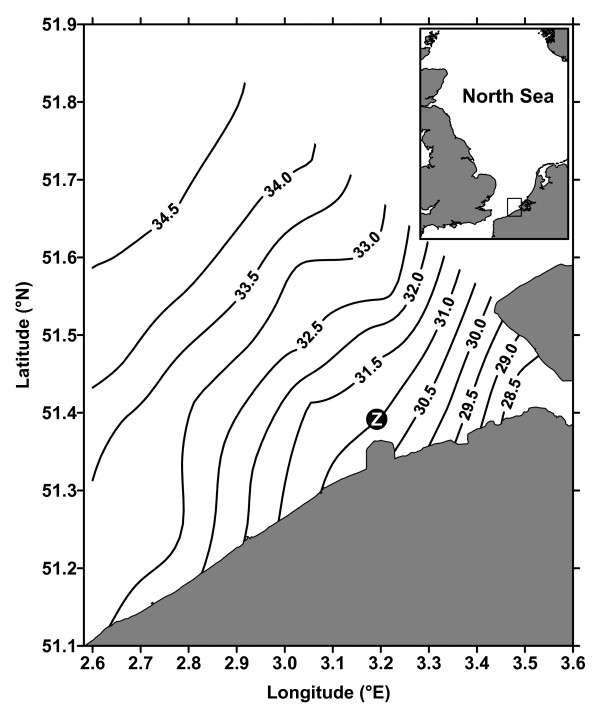
Position of the fixed station (Z, 3.18°E 51.37°N) near the Zeebrugge harbor and the climatological sea surface salinity distribution based on 160 cruises carried from 1995 to 2004.

Here, we report seasonal and inter-annual variations of NEP in the Scheldt estuarine plume (Fig. 1) estimated from a dissolved inorganic carbon (DIC) mass balance approach. This approach has previously provided robust estimates compared to incubation techniques in several coastal environments such as the Randers Fjord [12], the Scheldt inner estuary [13] and the Bay of Palma [17].

## Results and discussion

A wide range of techniques are used to estimate the metabolic status of coastal ecosystems, and each relies on one or several assumptions, and covers different spatial and temporal scales as reviewed by Gazeau et al. [12]. Integrative methods based on mass balance approaches of relevant biogeochemical variables (DIP, O_2_, DIC, organic carbon) have been recommended for estimation of metabolic performances in dynamic and complex coastal environments [12]. NEP can be established from a box model approach, by balancing the DIC inputs and outputs [e.g., [12,13,17-19]]. However, this method requires the knowledge of water flows that are typically highly variable in coastal environments, and tends to introduce a large uncertainty that is difficult to quantify. A more simple approach relies on the temporal variation of DIC, whereby:

$$\text{NEP}=-\frac{{\text{DIC}}_{2}-{\text{DIC}}_{1}}{\Delta \text{t}}\cdot \text{d}-\frac{{\text{FCO}}_{22}+{\text{FCO}}_{21}}{2}$$

where DIC_1 _and DIC_2 _are DIC values from 2 consecutive cruises, FCO_21 _and FCO_22 _are the air-sea CO_2 _fluxes (FCO_2_) from 2 consecutive cruises, Δt is the time interval between 2 consecutive cruises, and d is the depth of the mixed layer depth.

Such an approach is suited for permanently well-mixed systems such as the Belgian coastal zone (BCZ), as knowledge of the mixed layer depth is not required. This method relies on the assumption that the production and degradation of organic matter, and air-sea CO_2 _exchange are the main drivers of CO_2 _dynamics (and that other processes such as CaCO_3 _production/dissolution are negligible). Such an assumption holds true in the BCZ based on current understanding of CO_2 _dynamics in this region [9,20-24]. The major caveat of the method is the assumption that the net advective input/output of CO_2 _is constant between two steps of the computation. This source of uncertainty can be assumed minimal in the present case, because for time steps of the computations lower than the water residence time, the invariance of CO_2 _advective inputs/outputs can be assumed constant. The average time step of the computations was 21 d for an average water residence time of 60 d [25].

**Figure 2:**
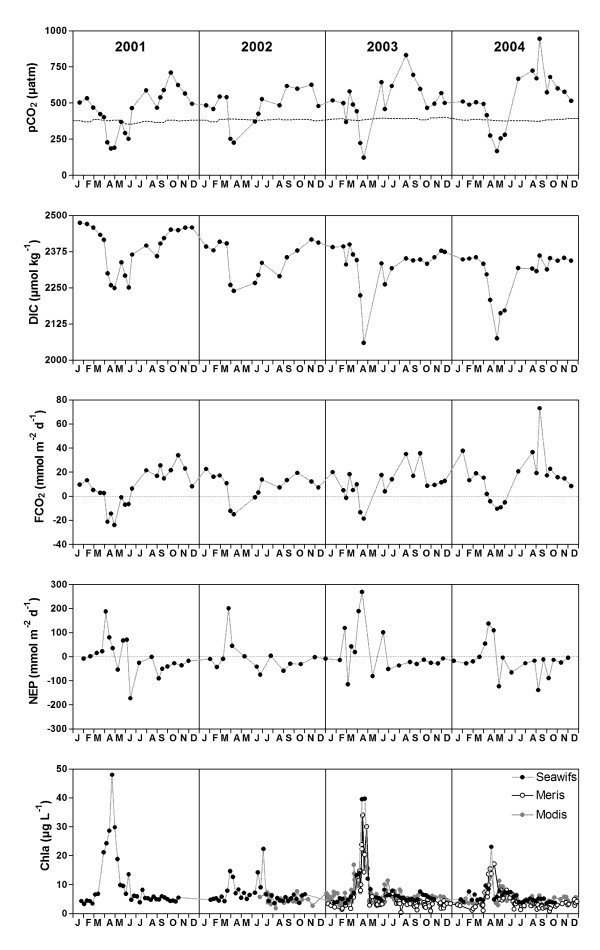
**Time series of the partial pressure of CO_2 _(pCO_2_), dissolved inorganic carbon (DIC), air-sea CO_2 _flux (FCO_2_) and net ecosystem production (NEP) in the Scheldt plume at a fixed station (3.18°E 51.37°N) near the Zeebrugge harbor, and of remote sensed chlorophyll-a concentration from the Sea-viewing Wide Field-of-view (SeaWiFS), Medium Resolution Imaging Spectrometer (MERIS) and Moderate Resolution Imaging Spectroradiometer (MODIS) sensors in a box (2.6–3.6°E; 51.1–51.5°N) corresponding to the average location of the Scheldt plume[e.g.,**[20,21]**].**

Figure 2 shows the time series of the partial pressure of CO_2 _(pCO_2_), DIC, FCO_2 _and NEP obtained at a reference station close to Zeebrugge harbor (Fig. 1) that is representative of seasonal pCO_2 _dynamics of the Scheldt estuarine plume [20,21]. Surface waters are under-saturated in CO_2 _with respect to atmospheric equilibrium during the spring bloom; during the rest of the year, surface waters are over-saturated in CO_2 _with respect to atmospheric equilibrium, more markedly in late summer than in fall and winter. A very strong seasonal draw-down of DIC is observed with a decrease of DIC from winter-time to spring-time of ~220 μmol kg^-1 ^in 2001 and 2002, and maximal value of ~340 μmol kg^-1 ^in 2003. From late spring to summer, DIC values increase due to the degradation of the organic carbon produced during the spring bloom. A transient and strongly autotrophic period occurs in spring that corresponds to minimal pCO_2 _and DIC values and that peaks in slightly different months from year to year (early April in 2001 and 2002, mid April in 2003 and 2004) with different amplitudes (maximal NEP values ranging from 269 mmol m^-2 ^d^-1 ^in 2004 to 134 mmol m^-2 ^d^-1 ^in 2003). The timing of the strongest net autotrophy is well correlated with the peak of remote sensed chlorophyll-a concentration (Fig. 2). This strongly autotrophic period can be attributed to the *Phaeocystis *bloom which occurs systematically in the BCZ at this time of year [e.g., [22,25-28]]. A significant diatom bloom preceding the *Phaeocystis *bloom is apparent from our NEP estimates only in February 2003. The *Phaeocystis *bloom is followed by a heterotrophic period after which an increase in NEP is observed. This increase in NEP leads to a net autotrophic event in early summer 2001 and 2003, and to a balanced metabolic status in 2002 and 2004. This increase in NEP can be ascribed to the summer diatom bloom that is known to be very variable in timing and amplitude in the BCZ [26-28]. Late summer is characterized by net heterotrophy that decreases during fall. A nearly balanced metabolic status is observed in winter.

**Figure 3:**
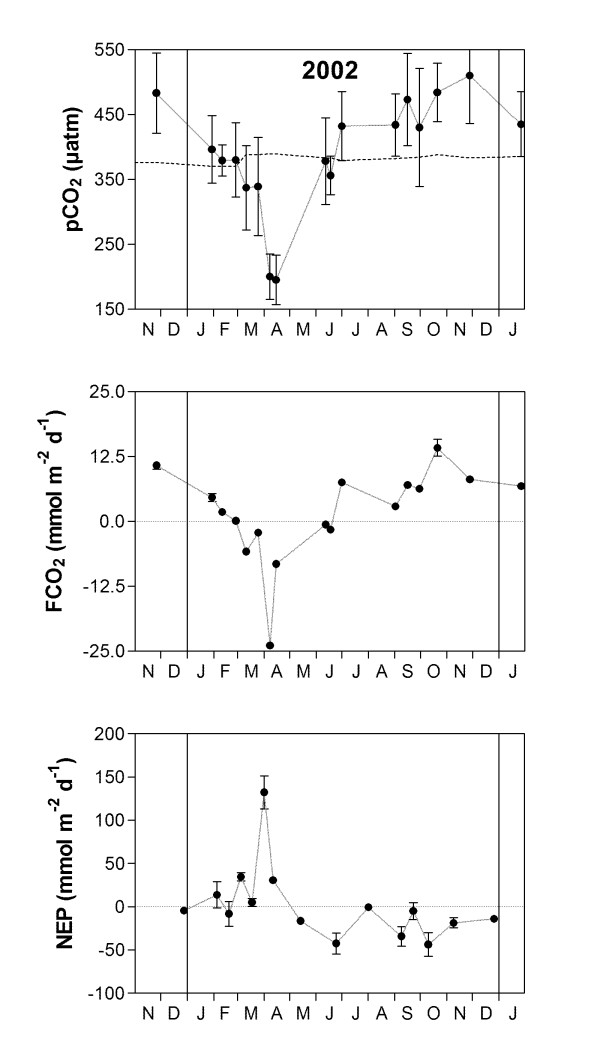
**Variation of the partial pressure of CO_2 _(pCO_2_), air-sea CO_2 _flux (FCO_2_) and net ecosystem production (NEP) in 2002, based on high spatial coverage surveys of the Scheldt plume (refer to**[23]**).** The Scheldt plume is defined as the area with a salinity < 34 [21]. Error bars correspond to the standard deviation on the mean values, reflecting the spatial variability of variables rather than measurement or computation uncertainties.

A pCO_2 _data-set of spatial surveys that covers the whole BCZ and satisfactorily captures the seasonal and spatial variability was obtained in 2002 [23]. This data-set consists of 17 cruises that cover the whole BCZ (2.3–3.6°E; 51.1–51.9°N); data in the Scheldt estuarine plume were extracted based on salinity (values < 34) [21], interpolated and averaged. This allows calculation of mean values for the whole Scheldt plume taking into account the spatial variability, which is not included in a fixed station approach. This allows verification that the NEP values computed from the fixed reference station are representative of the whole Scheldt plume. NEP values computed from the pCO_2 _survey data-set obtained in 2002 (Fig. 3) are consistent in timing and amplitude with those computed from the fixed reference station data-set (Fig. 2). On an annual scale, the NEP value computed from the pCO_2 _survey data-set obtained in 2002 is -3.1 ± 0.2 mol m^-2 ^yr^-1 ^in agreement with the value computed from the fixed reference station data-set for the same year (-3.8 ± 0.2 mol m^-2 ^yr^-1^, Table 1).

**Table 1:** Average Scheldt river fresh water discharge (Q) from January and December of the previous year, flux of dissolved inorganic nitrogen (FDIN) from the Scheldt river, flux of total inorganic phosphorous (FP_tot_) from the Scheldt river, winter-time DIN and PO_4_^2- ^concentrations in the Belgian coastal zone and annual averages of the partial pressure of CO_2 _(pCO_2_), air-sea gradient of pCO_2 _(ΔpCO_2_), air-sea CO_2 _flux (FCO_2_) and net ecosystem production (NEP) at a fixed station in the Scheldt plume near the Zeebrugge harbor.

	**Q ** **(m^3 ^s^-1^)**	**FDIN ** **(10^6 ^mol d^-1^)**	**FPtot ** **(10^6 ^mol d^-1^)**	**DIN ** **(μM)**	**PO_4_^2- ^** **(μM)**	**pCO_2 _** **(μatm)**	**ΔpCO_2 _** **(μatm)**	**FCO_2 _** **(mol m^-2 ^yr^-1^)**	**NEP ** **(mol m^-2 ^yr^-1^)**
2001	348	15	0.67	55.9	1.7	481	107	3.6	-4.2 ± 0.2
2002	302	12	0.41	45.2	1.3	480	97	3.2	-3.8 ± 0.2
2003	393	15	0.68	58.2	1.7	527	136	4.6	2.4 ± 0.1
2004	210	9	0.56	39.6	2.1	533	153	6.6	-5.7 ± 0.2

Fortnightly fresh water discharge data were provided by the Ministerie van de Vlaamse Gemeenschap Afdeling Waterbouwkundig Laboratorium en Hydrologisch Onderzoek. FDIN and FPtot were computed from average nutrient concentrations from January and December of the previous year from the Netherlands Institute of Ecology, Centre for Estuarine and Marine Ecology database and average Q from January and December of the previous year. Winter-time DIN and PO_4_^2- ^concentrations were obtained from the Management Unit of the North Sea Mathematical Models monitoring data-base for December of the previous year at salinity 31. The error on the NEP estimates was evaluated by propagating an error on pCO_2 _of ± 3 μatm on the DIC and air-sea CO_2 _flux computations.

On an annual scale the Scheldt river plume behaved as a net heterotrophic system in 2001, 2002 and 2004, but behaved as a net autotrophic system in 2003 (Table 1). Using a simple organic matter input/output budget, Borges and Frankignoulle [21] showed previously that the annual emission of CO_2 _to the atmosphere is only partly due to the input of CO_2 _from the Scheldt inner estuary and that net heterotrophy of the Scheldt estuarine plume is also important. The net heterotrophy of the Scheldt river plume must be sustained by external inputs of organic carbon that could originate from the Belgian coast and/or from the Scheldt inner estuary. Based on the input of organic matter from the Scheldt inner estuary reported by Soetaert and Herman [29] and a Scheldt plume surface area ranging between 2000 and 800 km^2 ^[20], we computed a potential organic matter degradation ranging between 0.3 and 0.6 mol m^-2 ^yr^-1^. Wollast [30] provides a higher estimate of the input of organic matter from the Scheldt inner estuary that can sustain a potential organic matter degradation ranging between 0.8 and 2.0 mol m^-2 ^yr^-1^. Finally, Wollast [31] estimated the input of organic carbon from the Belgian coast that can sustain a potential organic matter degradation ranging between 0.7 and 1.8 mol m^-2 ^yr^-1^. The potential degradation of these inputs of allochtonous organic matter from the Scheldt inner estuary and the Belgian coast are of the same order of magnitude as the annual NEP values we computed (Table 1).

The much stronger springtime NEP observed in 2003 compared to the other years is consistent with the remote sensed chlorophyll-a concentration from Medium Resolution Imaging Spectrometer (MERIS) and Moderate Resolution Imaging Spectroradiometer (MODIS) sensors showing that the peak springtime chlorophyll-a concentration was higher in 2003 than in 2004 (Fig. 2). It is known that the quality of satellite chlorophyll-a data may be suspect in coastal regions because of masking of phytoplankton absorption by absorption from coloured dissolved organic matter and/or non algal particles. For the region considered here such effects give a detection limit of about 3–5 μg L^-1 ^for chlorophyll-a concentration for the Sea-viewing Wide Field-of-view (SeaWiFS) and MODIS sensors, and possibly a lower limit for MERIS. This is seen here as a background (artificial) concentration at the level of this detection limit. However, the high phytoplankton biomass in spring are detected every year quite coherently by all three sensors, despite different atmospheric correction, overpass time and chlorophyll-a retrieval algorithms, giving confidence in the satellite detection of these blooms. SeaWiFS data suggest that peak springtime chlorophyll-a concentration was also higher in 2003 compared to 2002 (Fig. 2).

**Figure 4:**
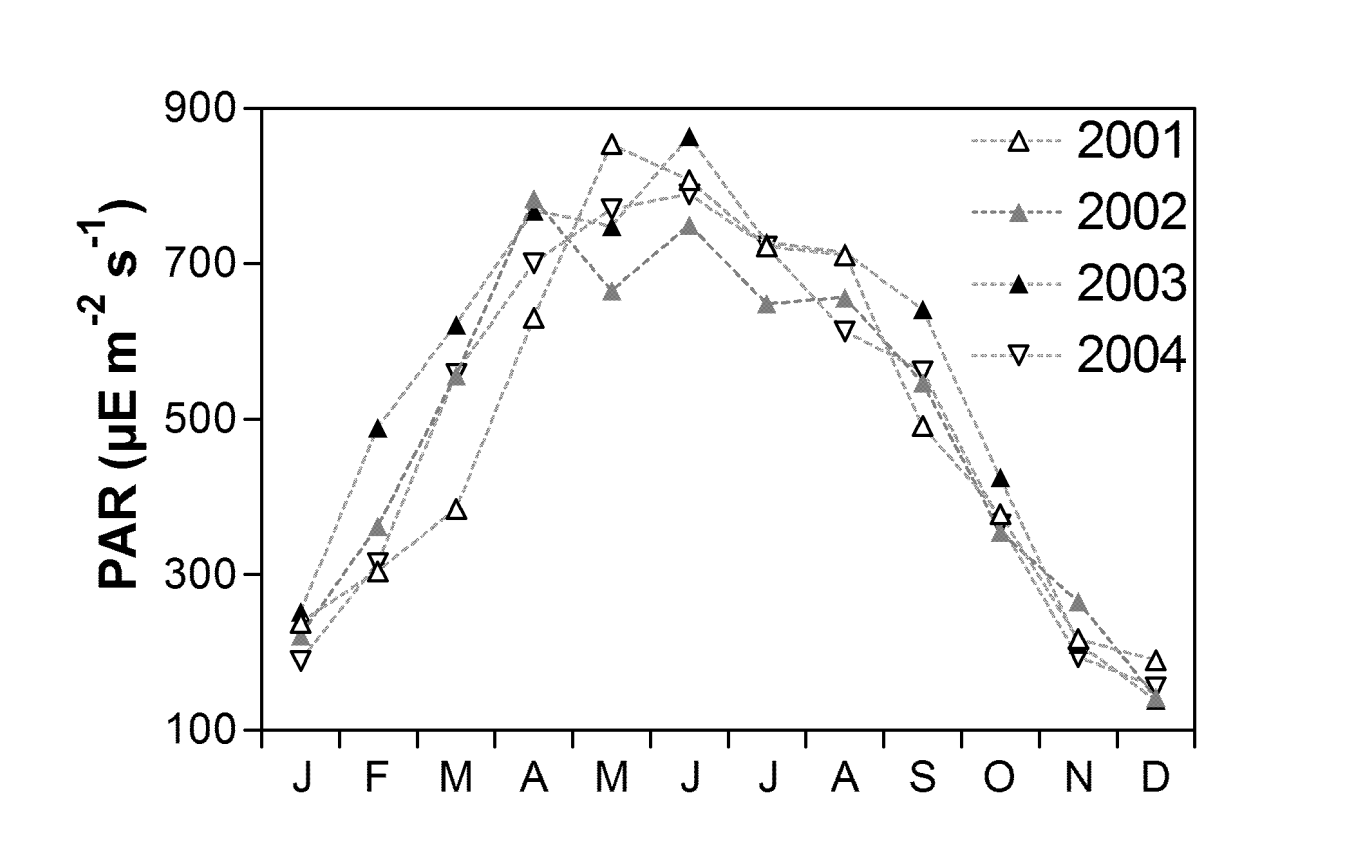
Monthly incoming photosynthetic active radiation (PAR) at Ostende from 2001 to 2004.

The much stronger springtime NEP observed in 2003 and annual net autotrophy observed in 2003 compared to the other years could be due to a combination of two processes. Wintertime freshwater discharge was stronger in 2003 than in the other years (Table 1). We used the average value of freshwater discharge in January and in December of the previous year, since the freshwater residence time in the Scheldt inner estuary ranges between 30 and 90 d [32]. Hence, the freshwater discharges during these months are those that can be assumed relevant for the productive season (from February to April) in terms of nutrient inputs from the Scheldt inner estuary. We hypothesize that in 2003 there was a stronger input of nutrients compared to the other years, while the input of organic matter was similar to other years. In the Scheldt inner estuary, the input of nutrients from diffuse sources are dependent on freshwater discharge, while organic matter comes mainly from point sources independently of freshwater discharge. This would lead to a stronger GPP in 2003 from the additional nutrient inputs, while allochtonous organic carbon inputs would sustain a similar level of heterotrophy as in the other years. We roughly evaluated the flux of dissolved inorganic nitrogen (FDIN) and of total phosphorous (FP_tot_) from the Scheldt river to the Scheldt estuarine plume (Table 1). In the BCZ, *Phaeocystis *is overwhelming more important than diatoms in terms of phytoplanktonic biomass and GPP [e.g., [22,25-28]], hence the computation of nutrient fluxes was not extended to silicate. FDIN values in 2003 were higher than in 2001 and 2004, while FP_tot _values in 2003 were higher than in 2002 and 2004 but similar to those of 2001. In the Scheldt estuarine plume, primary production during the spring phytoplankton bloom is strongly limited by P_tot _rather than by DIN [27,33]. Hence the higher FP_tot _values in 2003 could explain the higher NEP values during spring 2003 compared to 2002 and 2004. This is confirmed by the winter-time nutrient concentrations at salinity 31 in the BCZ: winter-time DIN concentrations were higher in 2003 than in 2001, 2002 and 2004; winter-time PO_4_^2- ^concentrations were higher in 2003 than in 2002 (Table 1). Also, incoming photosynthetically active radiation (PAR) was more favourable in 2003 during the productive months (February, March and April, Fig. 4), and could also explain the marked autotrophy associated to the phytoplankton bloom in 2003. Despite the fact that the Scheldt river plume was net autotrophic in 2003, it still acted as a net source of CO_2 _to the atmosphere (Table 1). This confirms that a fraction of this CO_2 _emission is sustained by the inputs of CO_2 _from the Scheldt inner estuary [21,23]. This fraction is expected to increase with increasing freshwater discharge.

The stronger annual heterotrophy in 2004 than in 2001 and 2002 could be due to a transient accumulation of part of the excess organic matter produced in 2003, since FPtot and winter-time PO_4_^2- ^concentrations were actually higher in 2004 than in 2002. The water residence time in the BCZ is highly variable but can be as long as 216 d [27] and is assumed to be on average 60 d [25]. Hence, we hypothesize that part of the non-steady accumulation of organic matter from 2003 to 2004 occurred in the sediments. Sedimentation of organic matter is important in the BCZ, representing about 20% of annual GPP [25], and gives bottom sediments that are exceptionally rich in organic carbon compared to the rest of the North Sea [30,34].

## Conclusion

The present work highlights the potential of using pCO_2 _data to derive detailed seasonal estimates of NEP in highly dynamic coastal environments, and to determine potential inter-annual variability of NEP due to natural climatic oscillations or due to changes in anthropogenic impacts. On a longer term, such an approach should also allow estimation of decadal changes in NEP that could be used as an indication of the effectiveness of nutrient control policies for reducing eutrophication of coastal waters.

## Methods

Automated measurements of pCO_2 _have been obtained since September 2000 on all the cruises carried out by the research vessel *Belgica*. A non-dispersive infrared gas analyzer (IRGA, Li-Cor^®^, Li-6262) and an equilibrator were used to measure the pCO_2 _(for details on design and performance tests refer to [35]). The IRGA was calibrated weekly using pure nitrogen (Air Liquide Belgium) and two gas mixtures with a CO_2 _molar fraction of 366 and 810 ppm (Air Liquide Belgium) that were calibrated against National Oceanic and Atmospheric Administration standards of a CO_2 _molar fraction of 361 and 774 ppm. The temperature at the outlet of the equilibrator was monitored with a platinum resistance thermometer (PT100, Metrohm^®^). The pCO_2 _values were corrected for the temperature difference between *in-situ *seawater and water in the equilibrator using the algorithm given by Copin-Montégut [36,37]. The overall accuracy of pCO_2 _measurements is estimated to be better than ± 3 μatm. Salinity and temperature were measured using a SeaBird^® ^SBE21 thermosalinograph. Salinity, temperature and pCO_2 _were sampled from the seawater supply of the ship (pump inlet at a depth of 2.5 m) and logged at a 1 min frequency.

The FCO_2 _was computed from the air-sea pCO_2 _gradient (ΔpCO_2 _= pCO_2_sea - pCO_2_air), the solubility coefficient of CO_2 _(α), and the gas transfer velocity (*k*) according to:

FCO_2 _= α *k *ΔpCO_2_

The *k *values were computed using hourly wind speed values from the Vlakte van de Raan meteorological station (3.24°E 51.52°N) provided by the Royal Netherlands Meteorological Institute, and the *k*-wind parameterization given by Nightingale et al. [38], established in the Southern Bight of the North Sea, close to our study area. Monthly values of atmospheric pCO_2 _data obtained at station Kollumerwaard in the Netherlands (6.17°E 53.20°N) were provided by the Dutch National Air Quality Monitoring Network. Atmospheric pCO_2 _data were converted into pCO_2 _in wet air according to Dickson and Goyet [39].

Total alkalinity (TA) was computed according to:

TA = 3929 - 46.156*SSS   (r^2 ^= 0.872, p < 0.0001)

where SSS is sea surface salinity, and TA is in μmol kg^-1^, established from 742 measurements in surface waters (salinity range 19.5–35.4) from 26 cruises carried out in the BCZ from 1996 to 2001 [[20,21], Borges unpublished, Schiettecatte unpublished]. TA was measured using the Gran electrotitration method, with an estimated accuracy of ± 3 μmol kg^-1^. DIC was computed from pCO_2 _measurements and TA estimates from SSS, using the carbonic acid constants of Mehrbach et al. [40] refitted by Dickson and Millero [41].

Global solar radiation (GR) was recorded at the Ostende meteorological station (51.15°N 02.54°E) by the Institut Royal de Météorologie de Belgique, and were converted into daily-averaged PAR using the following empirical relationship [42]:

$$\text{PAR}=\frac{12.14*\text{GR}}{48}$$

where GR is in J cm^-2 ^d^-1 ^and PAR is in μmolE m^-2 ^s^-1^

Level-3 SeaWiFS chlorophyll-a concentration data were extracted from the Ocean Color Time-Series Online Visualization and Analysis web site . Level-2 MODIS chlorophyll-a concentration data were derived from the L1A MODIS data, distributed by NASA Goddard Space Flight Center Ocean Color group . The L1A radiance data measured by the sensor at the top of atmosphere are processed using the SeaWiFS Data Analysis System software with the atmospheric correction of Ruddick et al. [43] to obtain atmospherically corrected radiances. These are then converted to chlorophyll-a concentrations using the OC3 algorithm [44]. Two chlorophyll-a parameters are included in MERIS level-2 products . The "algal pigment index 1" is computed using a ratio of water reflectances at blue and green bands [45,46] and represents chlorophyll-a concentration for oceanic case 1 waters. The "algal pigment index 2" is designed to represent chlorophyll-a concentration for coastal case 2 waters and computed using a neural-network multiband inversion technique [47]. The MERIS chlorophyll-a concentration used in this study was taken either from the algal pigment index 2 if the MERIS case 2 water flag was set or algal pigment index 1 otherwise. These chlorophyll-a data were removed if the product confidence flag was raised, thus excluding unreliable data. It is expected that the MERIS chlorophyll-a data will be more reliable in this region than those from SeaWiFS and MODIS because the case 2 algorithm is better suited to waters with high yellow substance absorption.

## Authors' contributions

AVB conceived and designed the study, coordinated and drafted the manuscript. KR provided remote sensed chlorophyll-a data. LSS carried out the field pCO_2 _measurements. BD helped to conceive the study and draft the manuscript. All authors read and approved the final manuscript.
